# Folgen der COVID-19-Pandemie: Gibt es Risikogruppen für ein verringertes subjektives Wohlbefinden nach dem ersten Lockdown?

**DOI:** 10.1007/s00103-023-03737-w

**Published:** 2023-07-20

**Authors:** Emily Finne, Oliver Razum

**Affiliations:** Fakultät für Gesundheitswissenschaften, School of Public Health, Arbeitsgruppe Epidemiologie & International Public Health, Universität Bielefeld, PF 10 01 31, 33501 Bielefeld, Deutschland

**Keywords:** Coronavirus, Ungleichheiten, Subjektive Gesundheit, Panelstudie, Intersektionalität, Coronavirus, Inequalities, Subjective health, Panel study, Intersectionality

## Abstract

**Hintergrund:**

Maßnahmen zur Eindämmung von COVID-19 führten zu Belastungen, die gesundheitliche Ungleichheiten verstärkt haben. Wir untersuchen, inwiefern sich Risikogruppen für ein reduziertes subjektives Wohlbefinden nach Eintreten des Lockdowns 2020 identifizieren lassen. Dabei berücksichtigen wir im Rahmen eines intersektionalen Ansatzes auch mögliche Wechselwirkungen verschiedener sozialer Gruppierungsmerkmale.

**Methode:**

Analysiert wurden Daten des Sozio-oekonomischen Panels (SOEP) aus den Jahren 2018–2020. Insgesamt 16.000 Fälle mit Angaben zu Wohlbefindensänderungen (SF-12-Scores sowie Einzelindikatoren) wurden in die Auswertung einbezogen. Zur Identifikation von Gruppen mit unterschiedlicher Entwicklung im Wohlbefinden verwenden wir das Klassifikationsverfahren Random Forests. Zur inhaltlichen Interpretation stellen wir ergänzend Ergebnisse aus einem Regressionsmodell mit sozialen und gesundheitlichen Aspekten als Prädiktoren dar.

**Ergebnisse:**

Demografische und soziale Merkmale erklärten nur einen sehr geringen Teil der Veränderungen im subjektiven Wohlbefinden (R^2^ = 0,007–0,012) und ließen keine Abgrenzung homogener Risikogruppen zu. Obwohl einige signifikante Prädiktoren in den Regressionsmodellen gefunden wurden, waren entsprechende Effekte überwiegend gering. Neben dem Ausgangszustand im Wohlbefinden vor Pandemiebeginn trugen v. a. das Vorliegen chronischer Erkrankungen und Behinderungen zur Erklärung des Wohlbefindens bei.

**Diskussion:**

Die aktuell vorliegenden Daten ermöglichen keine klare Identifikation von Risikogruppen für Einbußen im Wohlbefinden im ersten Jahr der COVID-19-Pandemie. Der Gesundheitszustand vor Pandemiebeginn scheint für kurzfristige Veränderungen im subjektiven Wohlbefinden bedeutsamer zu sein als soziodemografische und -ökonomische Kategorisierungsmerkmale.

**Zusatzmaterial online:**

Zusätzliche Informationen sind in der Online-Version dieses Artikels (10.1007/s00103-023-03737-w) enthalten.

## Abstract (en)

**Background:**

Measures to contain COVID-19 have created burdens that have widened health inequalities. We examine the extent to which risk groups for reduced subjective well-being can be identified after the 2020 lockdown. In doing so, we also consider possible interactions of different social grouping characteristics as part of an intersectional approach.

**Method:**

Socio-Economic Panel (SOEP) data from the years 2018–2020 were analyzed. A total of 16,000 cases with information on changes in well-being (SF-12 scores and individual indicators) were included in the analyses. We use the classification method “random forests” to identify groups with different trends in well-being. For the interpretation of the content, we also present results from a regression model with social and health aspects as predictors.

**Results:**

Demographic and social characteristics explained only a very small part of the changes in subjective well-being (R^2^ = 0.007–0.012) and did not allow for the differentiation of homogeneous risk groups. Although some significant predictors were found in the regression models, the corresponding effects were mostly small. In addition to the initial state of well-being before the pandemic began, the presence of chronic illnesses and disabilities in particular contributed to the explanation of subjective well-being.

**Discussion:**

The currently available data do not allow a clear identification of risk groups for losses in well-being in the first year of the COVID-19 pandemic. Health status before the onset of the pandemic appears to be more important for short-term changes in subjective well-being than socio-demographic and socio-economic categorization characteristics.

## Hintergrund

Sozial ungleich verteilte gesundheitliche Auswirkungen der COVID-19-Pandemie ließen sich international und national vielfach nachweisen [1–5]. Hinsichtlich Infektionsraten, Hospitalisierung und Mortalität besonders betroffen waren z. B. Menschen aus sozial deprivierten Regionen [6, 7], mit geringerem Bildungsgrad [8], Migrations- oder Fluchterfahrung [9, 10] sowie Arbeitslose [11]. Als anfälliger für eine Infektion und schwere Krankheitsverläufe erwiesen sich außerdem ältere Menschen und Personengruppen mit Vorerkrankungen sowie starkem Übergewicht [12].

Auch Maßnahmen zur Eindämmung der Pandemie (nicht-pharmakologische Interventionen – NPI, wie Schulschließungen und Kontaktbeschränkungen) können sich über ihre wirtschaftlichen und sozialen Konsequenzen auf die Gesundheit verschiedener Bevölkerungsgruppen unterschiedlich auswirken und so gesundheitliche Ungleichheiten verstärken. Dabei gibt es klare Hinweise, dass Belastungen durch NPI sich sozial ungleich verteilt haben [13–16]. Stärker beeinträchtigt wurden z. B. Frauen, Menschen in prekärer Beschäftigung und mit geringer Qualifikation oder solche mit Migrationserfahrung. Aber auch Bevölkerungsgruppen, welche i. d. R. nicht zu den klassischen benachteiligten Gruppen gezählt werden, wie Selbstständige und Künstler:innen, erwiesen sich als stärker durch wirtschaftliche Folgen der NPI betroffen (z. B. [17]). Solche ungleich verteilten Belastungen können wiederum (als indirekte Pandemiefolgen) gesundheitliche Ungleichheiten verstärken. Dabei ist anzunehmen, dass sich indirekte Gesundheitsfolgen in Krisen zunächst im subjektiven Wohlbefinden widerspiegeln, bevor sie sich bei andauernder Belastung in manifesten Erkrankungen niederschlagen. Subjektive Gesundheitsdimensionen wie die gesundheitsbezogene Lebensqualität, inkl. des psychischen und körperlichen Wohlbefindens, stellen wichtige Aspekte der Gesundheit dar und scheinen damit besonders geeignet, kurz- bis mittelfristige gesundheitliche Auswirkungen der pandemiebedingten Situation abzubilden. Wir verwenden hier den Begriff Wohlbefinden als Oberbegriff für verschiedene Dimensionen der subjektiven Gesundheit und Lebensqualität.

In der Corona-Health-App-Studie des Robert Koch-Instituts (RKI) wurde im zweiten Halbjahr 2020 ein geringeres Wohlbefinden berichtet bei Menschen mit geringerem Bildungsgrad, in jüngeren Altersgruppen, bei nicht regulär arbeitenden Befragten sowie beim weiblichen Geschlecht [18]. Weitere Studien zeigten Anstiege in negativen Emotionen wie Einsamkeitsgefühlen, v. a. bei Jüngeren [19], sowie Depressivität und Ängstlichkeit [20, 21], z. T. mit deutlichen sozialen Gradienten. International waren Ausgangsbeschränkungen mit einer Reduktion der Lebenszufriedenheit verbunden [22].

Die genannten Befunde zeigen eine größere Betroffenheit einiger Bevölkerungsgruppen durch direkte und indirekte Folgen der Pandemie. Um gesundheitlichen Ungleichheiten oder ihrer Verstärkung infolge der Pandemiesituation entgegenzuwirken, sollten besonders gefährdete Bevölkerungsgruppen gezielt durch unterstützende Maßnahmen adressiert werden [23]. Dies setzt zunächst die Identifikation solcher Risikogruppen voraus. Dabei wurden Kategorisierungsmerkmale wie Geschlecht, sozioökonomischer Status und Alter bislang i. d. R. einzeln untersucht. Das Zusammenwirken verschiedener Benachteiligungsdimensionen wurde erst wenig betrachtet.

Im Mittelpunkt des Konzeptes „Intersektionalität“ stehen Benachteiligungen, welche sich aus der Verschränkung verschiedener sozialer Diversitätsmerkmale ergeben können. Der Begriff wurde von Kimberlé Crenshaw [24] geprägt und bezieht sich ursprünglich auf die Marginalisierung schwarzer Frauen durch die getrennte Betrachtung der Diskriminierungsfaktoren Ethnie und Geschlecht. Er lässt sich jedoch auf weitere marginalisierende Faktoren, wie z. B. den Sozialstatus, Behinderungen, chronische Erkrankungen oder die sexuelle Orientierung, beziehen. Erfahrungen mit der Zugehörigkeit zu verschiedenen sozialen Kategorien beeinflussen sich gegenseitig und es wird angenommen, dass hier Dynamiken über additive Effekte hinaus wirken. Kategorien können als Personen- und soziale Kontextmerkmale betrachtet werden, indem sie die Identität formen und durch den sozialen Kontext hergestellt werden [25].

Es wurde gefordert, Aspekte der Intersektionalität auch in quantitativen Studien zur Bevölkerungsgesundheit stärker zu thematisieren [26–29]. Im Gegensatz zu Auswirkungen der Pandemie hinsichtlich einzelner sozialer Kategorisierungen ist ihr Zusammenwirken jedoch erst wenig untersucht. Eine Ausnahme bilden z. B. Auswertungen der SOEP-CoV-Studie, in der die Wechselwirkung von Geschlecht und beruflicher Selbstständigkeit betrachtet wurde [17, 30]. Auch international finden sich in der Literatur zu COVID-19 vorwiegend Aufrufe zu einer stärkeren Berücksichtigung intersektionaler Aspekte (z. B. [31–34]), während empirisch bisher eher spezifische Risikogruppen exemplarisch betrachtet wurden (z. B. [35–37]). Unklar bleibt dabei, welche (gerade mehrfach marginalisierten) Bevölkerungsgruppen in Bezug auf verschiedene gesundheitliche Outcomes besonders betroffen waren. Hier scheinen Erkenntnisse aus unterschiedlichen Kulturkreisen aufgrund der sehr verschiedenen Bedingungen und Auswirkungen der Pandemie zudem nur bedingt vergleichbar zu sein.

Ziel unseres Beitrags ist die Untersuchung möglicher Diversitätskonstellationen für eine besondere Beeinträchtigung im Wohlbefinden durch die Pandemie. Die Herausforderung liegt in der explorativen Identifikation von Risikogruppen, ohne im Vorweg die Betrachtung auf bereits bekannte Konstellationen einzuschränken. Dabei beziehen wir als potenzielle Gruppierungsmerkmale v. a. soziale Dimensionen ein, die im Kontext von COVID-19 bereits mit direkten oder indirekten gesundheitlichen Folgen in Zusammenhang gebracht wurden (s. o). Durch unser Vorgehen berücksichtigen wir insbesondere auch die Möglichkeit von Intersektionen, d. h. Kombinationen der betrachteten Merkmale bei der Definition von Risikogruppen, ohne diese jedoch vorzugeben.

## Methode

Die Datenbasis unserer Auswertungen bildet das *Sozio-oekonomische Panel (SOEP) *in der Version 37 mit Daten bis einschließlich der Erhebungswelle 2020 [38]. Dabei handelt es sich um eine deutschlandweite Repräsentativbefragung, welche seit 1984 jährlich durchgeführt wird und längsschnittliche Daten zu einer Vielzahl wirtschaftlicher, sozialer und auch gesundheitlicher Indikatoren liefert. Wir berücksichtigen alle Befragten, bei denen Wohlbefindensaspekte vor und nach Lockdownbeginn erhoben wurden.

Hinsichtlich des Wohlbefindens betrachten wir individuelle Veränderungen zwischen der letzten Erhebung vor Beginn des ersten Lockdowns (23.03.2020) und der ersten Erhebung danach. Als potenzielle Prädiktoren berücksichtigen wir jeweils die Ausprägung zum letzten Erhebungszeitpunkt vor dem Lockdown (2018/2019 bis März 2020). Da es sich um eine Sekundäranalyse bereits erhobener SOEP-Daten handelt, sind ethische Aspekte im Umgang mit Befragten für unsere Analysen nicht relevant.

### Maße

Als subjektive *Wohlbefindensmaße *betrachten wir die 2 Skalenwerte des Screeninginstruments „Short-Form-Health Survey“ (SF-12) zu mentalem und körperlichem Wohlbefinden. Diese basieren jeweils auf mehreren Items eines international vielfach erprobten Instruments. Die Scores wurden anhand der Werte der SOEP-Stichprobe 2004 normiert [39, 40]. Allerdings wurden die Ausgangswerte zuletzt 2018 erhoben, so dass Veränderungen weniger direkt auf die Pandemie zurückzuführen sein könnten. Daher beziehen wir 3 weitere, jährlich erhobene Indikatoren ein. Wir verwenden ein Item zum selbsteingeschätzten allgemeinen Gesundheitszustand sowie die Angaben zur allgemeinen Lebenszufriedenheit (als Maß für allgemeines Wohlbefinden) und zur Zufriedenheit mit der eigenen Gesundheit (bereichsspezifisches Wohlbefinden).

Als potenzielle *Gruppierungsmerkmale* einbezogen werden Variablen, die in der Literatur im Zusammenhang mit gesundheitlicher Ungleichheit allgemein sowie hinsichtlich COVID-19 diskutiert wurden. Darunter fallen neben demografischen und sozioökonomischen Merkmalen auch Angaben zu dem Vorliegen von Schwerbehinderung oder chronischen Erkrankungen sowie Übergewicht. Die einbezogenen Variablen sind in Tab. 1 beschrieben. Detaillierte Angaben zur Operationalisierung finden sich in den entsprechenden Veröffentlichungen zur Methodik im SOEP^1^.

**Table 1:** 

*Aspekte des subjektiven Wohlbefindens:*	
SF-12 (Erhebung 2018 und 2020):	Der SF-12 stellt ein kurzes Screeninginstrument zur Ermittlung der gesundheitsbezogenen Lebensqualität in verschiedenen Bereichen dar. Eigenschaften der deutschsprachigen (SOEP-)Version und die Berechnung werden in [39] und [40] dargestellt
Skala psychische Gesundheit (MCS)	Der Punktwert zur psychischen Gesundheit beinhaltet Angaben zu emotionaler Rollenfunktion, psychischem Wohlbefinden, negativem Affekt und sozialer Funktionsfähigkeit. Die Werte wurden anhand der SOEP-Stichprobe aus 2004 T-normiert (M = 50, SD = 10)
Skala körperliche Gesundheit (PCS)	Der Punktwert zur körperlichen Gesundheit setzt sich aus Angaben zu allgemeiner Gesundheitswahrnehmung, körperlicher Funktionsfähigkeit und Rollenfunktion sowie Schmerzen zusammen. Die Werte wurden anhand der SOEP-Stichprobe aus 2004 T-normiert (M = 50, SD = 10)
*Einzelindikatoren zum Wohlbefinden (Erhebung in den Jahren 2019 und 2020):*	
Allgemeine Lebenszufriedenheit (psychisches Wohlbefinden)	„Wie zufrieden sind Sie gegenwärtig, alles in allem, mit Ihrem Leben?“ Mit einer Antwortskala von 0 = ganz und gar unzufrieden bis 10 = ganz und gar zufrieden
Zufriedenheit mit der eigenen Gesundheit	„Wie zufrieden sind Sie mit Ihrer Gesundheit?“ Mit einer Antwortskala von 0 = ganz und gar unzufrieden bis 10 = ganz und gar zufrieden
Selbsteingeschätzter allgemeiner Gesundheitszustand	„Wie würden Sie Ihren gegenwärtigen Gesundheitszustand beschreiben?“ Antwortmöglichkeiten: 1 = sehr gut, 2 = gut, 3 = zufriedenstellend, 4 = weniger gut, 5 = schlecht; Für die Auswertungen wurden die Antwortkategorien umgepolt
*Potenziell relevante Merkmale für Vorhersagen und die Unterscheidung von Gruppen:*	
Alter	Berechnet aus Erhebungsjahr und Geburtsjahr
Geschlecht	Referenzkategorie = weiblich
Migrationshintergrund	Zur Ermittlung der Migrationsgeschichte werden im SOEP verschiedene Angaben kombiniert – basierend auf dem Geburtsland der Befragten und ihrer Eltern. Ein direkter Migrationshintergrund wird angenommen bei Geburt im Herkunftsland, ein indirekter Migrationshintergrund bei Geburt in Deutschland sowie Elternteil mit direkter Migrationserfahrung
Familienstand	„Wie ist Ihr Familienstand?“ Unter „verheiratet“ klassifiziert wurden Personen mit den Angaben „verheiratet“ sowie „eingetragene gleichgeschlechtliche Partnerschaft“
Alleinerziehend	Wurde angenommen, wenn im Haushalt neben angegebenen Kindern nur eine erwachsene Person lebt
Single-Haushalt	Bei einer Personenanzahl von „1“ für den Haushalt
Neue Bundesländer	Adresse des Haushalts in den neuen Bundesländern
Bildung nach CASMIN-Klassifikation	Angaben zum Schulabschluss, klassifiziert nach dem Schema der „Comparative Analysis of Social Mobility in Industrial Nations (CASMIN)“; dabei wurden die 3 Hauptgruppen unterschieden [51]
Chronische Erkrankung	„Leiden Sie seit mindestens einem Jahr oder chronisch an bestimmten Beschwerden oder Krankheiten?“Antwortoptionen: Ja/Nein
Übergewicht oder Adipositas	Erfragt wurden Körpergröße und -gewicht. Daraus berechnet wird der Body-Mass-Index (BMI) als Körpergewicht in kg/(Körpergröße in m)^2^. Die Werte wurden bei einem BMI ab 25 als Übergewicht und bei einem BMI ab 30 als Adipositas klassifiziert [52]. Die Referenzkategorie stellen Personen mit einem BMI unter 25 dar
Behinderung	„Sind Sie nach amtlicher Feststellung erwerbsgemindert oder schwerbehindert?“ Antworten: Ja/Nein, bei Ja: freie Angabe zu Grad der Behinderung; Als Behinderung klassifiziert wurde eine vorliegende Behinderung ab einem Grad von 30 %
Erwerbsstatus	„Üben Sie derzeit eine Erwerbstätigkeit aus? Was trifft für Sie zu?“ *Antwortoptionen:*Voll erwerbstätig - In Teilzeitbeschäftigung - In betrieblicher Ausbildung/Lehre oder betrieblicher Umschulung - Geringfügig oder unregelmäßig erwerbstätig - In Altersteilzeit mit Arbeitszeit Null - Im Freiwilligen Sozialen/Ökologischen Jahr, im Bundesfreiwilligendienst - nicht erwerbstätig Für die Auswertung wurden die Angaben zu 4 Kategorien zusammengefasst
Berufliche Selbstständigkeit	„In welcher beruflichen Stellung sind Sie derzeit beschäftigt?“ Die Antwortoption „Selbstständige (einschl. mithelfende Familienangehörige)“ wurde als Selbstständigkeit kodiert
Arbeitsstunden pro Woche	„(…) wie viel beträgt im Durchschnitt Ihre tatsächliche Arbeitszeit pro Woche einschließlich eventueller Überstunden?“ Antwort: freies Format
Nettoäquivalenzeinkommen	Ermittelt wird das monatliche Nettoeinkommen aus verschiedenen Angaben zu Einkünften im Haushalt, wobei die Personenanzahl mit modifizierten Äquivalenzgewichten ermittelt wird. Berechnet wird das Pro-Kopf-Einkommen im Haushalt. Eine Berechnungsvorschrift findet sich bei [53]

*MCS* Mental Component Score, *PCS* Physical Component Score, *SF-12* Short-Form-Health Survey, *SOEP* Sozio-oekonomisches Panel

### Beschreibung des statistischen Verfahrens und Vorgehens

Um Gruppen zu identifizieren, welche in ihrem subjektiven Wohlbefinden pandemiebedingt besonders beeinträchtigt wurden, haben wir das Klassifikationsverfahren Random Forests verwendet. Diese explorative Methode aus dem Bereich des maschinellen Lernens beruht auf Entscheidungsbäumen. Es soll die Vorhersage eines Outcomes anhand der vorhandenen Prädiktoren und ihres Zusammenwirkens optimiert werden [41, 42]. Das Verfahren scheint besonders geeignet, da automatisch auch Interaktionen zwischen Prädiktoren berücksichtigt werden, sofern diese zu einer Unterscheidung der Gruppen beitragen. Dies entspricht den propagierten Effekten von Intersektionalität. Das Verfahren wählt für die enthaltenen Entscheidungsbäume schrittweise Prädiktoren aus, welche zu möglichst unterschiedlichen Gruppen hinsichtlich des Outcomes führen. Je Gruppe wird dabei bei kontinuierlichen Outcomes für alle Mitglieder der Mittelwert vorhergesagt (s. ausführliche Beschreibung der Methode Random Forests im Onlinematerial).

Im Unterschied zu klassischen sozialwissenschaftlichen Methoden zielen Machine-Learning-Ansätze primär auf die Optimierung der Vorhersage ab und weniger auf eine inhaltliche Interpretierbarkeit der resultierenden Modelle. Random Forests geben inhaltliche Anhaltspunkte nur durch eine Rangfolge der Wichtigkeit einzelner Merkmale, liefern aber keine Charakterisierung unterschiedener Gruppen. Daher wählen wir ergänzend die Darstellung eines linearen Regressionsmodells, um Gruppenunterschiede über die Modellkoeffizienten abzubilden. Da es sich bei Random Forests um nicht-parametrische Modelle handelt und auch nicht-lineare Effekte einbezogen werden können, die Voraussetzungen des Regressionsverfahrens verletzen können, verwenden wir bei der Schätzung robuste Standardfehler.

Für die Erstellung der Random Forests haben wir das R‑Paket *ranger* und zur Vorbereitung der Daten *caret* genutzt [43]. 70 % der Fälle mit vorliegendem Outcome wurden jeweils als Trainingsdaten zur Optimierung des Modells verwendet.

Tuningparameter haben wir über die Prozedur *tuneRanger* unter Nutzung einer wiederholten 10fachen Kreuzvalidierung optimiert. Daraus haben wir die Anzahl je Schritt zu berücksichtigender Variablen, minimale Gruppengröße sowie den optimalen Stichprobenanteil übernommen und für jedes Outcome 1000 Bäume zusammengefasst. Das Kriterium für die Wichtigkeit einzelner Variablen bildete die Varianzerklärung. Dabei wird für jede Variable ermittelt, wie stark die unerklärte Varianz durch diese, gemittelt über alle Bäume, reduziert wird. Wichtige Variablen führen zu der größten Reduktion [41]. Zur Beurteilung der Modellgüte verwenden wir den mittleren quadratischen Vorhersagefehler (RMSE) sowie R^2^ für die erklärte Varianz.

Wir beschreiben die Effekte der Prädiktorvariablen im Rahmen von Regressionsmodellen mit den gleichen potenziellen Prädiktoren. Veränderungen im Wohlbefinden stellen wir über die für den jeweiligen Ausgangswert vor Lockdown adjustierten Outcomes dar (ANCOVA-Ansatz, s. [44]). Damit sind Gruppenunterschiede für bereits vor der Pandemie bestehende Unterschiede adjustiert. Weiterhin adjustiert wurde die seit Beginn des ersten Lockdowns vergangene Zeit. Als Maße der Modellgüte werden auch hier RMSE und erklärter Varianzanteil (R^2^) betrachtet. Um die Rolle der eingeschlossenen Kontrollvariablen (Ausgangswerte) zu berücksichtigen, haben wir zum Vergleich auch den erklärten Varianzanteil in den Differenzwerten ohne Adjustierung ermittelt. Alle numerischen Variablen wurden so kodiert, dass jeweils höhere Werte für eine höhere Merkmalsausprägung stehen. Kategoriale Variablen wurden dummykodiert.

Fehlende Werte in kategorialen Prädiktorvariablen wurden jeweils als eigene Kategorie kodiert. Bei kontinuierlichen Prädiktoren wurden sie über die Funktion *missRanger* [45, 46] imputiert, um eine deutliche Reduktion der Stichprobengröße zu vermeiden. Fehlende Angaben in den Outcomewerten wurden nicht ersetzt.

## Ergebnisse

### Deskription

Tab. 2 zeigt die deskriptiven Statistiken für die betrachteten Wohlbefindensmaße auf Grundlage aller einbezogenen Fälle. Dabei gehen wir von den nicht imputierten Werten aus und geben jeweils den Anteil fehlender Werte wieder.

**Table 2:** 

	Vor Lockdown: M (SD)	% Missing	Nach Lockdown: M (SD)	% Missing
SF-12 psychische Gesundheit	50,68 (10,00)	35,01	50,00 (10,07)	7,03
SF-12 körperliche Gesundheit	50,55 (9,86)	35,01	50,94 (9,81)	7,03
Lebenszufriedenheit	7,57 (1,70)	11,19	7,58 (1,66)	7,80
Zufriedenheit Gesundheit	7,09 (2,20)	11,29	7,11 (2,17)	7,74
Allg. Gesundheitszustand	3,61 (0,99)	9,63	3,61 (0,98)	7,73
*Differenzwerte:*				
∆ SF-12 psychische Gesundheit	–	–	−0,72 (10,85)	36,77
∆ SF-12 körperliche Gesundheit	–	–	−0,29 (8,53)	36,77
∆ Lebenszufriedenheit	–	–	0,00 (1,66)	14,31
∆ Zufriedenheit Gesundheit	–	–	0,00 (1,96)	14,17
∆ Allg. Gesundheitszustand	–	–	0,00 (0,85)	12,66

*∆* Differenz der Werte nach Lockdown minus Werte vor Lockdownbeginn, *M* arithmetisches Mittel, *SD* Standardabweichung, *SF-12* Short-Form-Health Survey, *% Missing* Anteil fehlender Angaben im Datensatz (bezogen auf die Ausgangsstichprobe von 16.000 Fällen)

### Ergebnisse zu Gruppenunterschieden

#### Random Forests

Die Anteile der durch die einbezogenen demografischen und sozialen Prädiktoren erklärten Varianz in Veränderungen des Wohlbefindens lagen für die verschiedenen Outcomes nur zwischen 0,4 % und 1,6 % (Tab. 3). Eine zuverlässige Vorhersage und damit auch eine klare Unterscheidung von homogenen Risikogruppen waren damit für keines der Outcomes möglich. Dennoch lässt sich die relative Wichtigkeit der Variablen bestimmen. Tab. 4 bildet die Rangfolgen der bedeutsamsten Prädiktoren in den Random Forests ab.

**Table 3:** 

**S**ubjektives Wohlbefinden → Koeffizienten (s. e.)	Psychische Gesundheit (SF-12)	Körperliche Gesundheit (SF-12)	Lebenszufriedenheit	Zufriedenheit mit Gesundheit	Allgemeiner Gesundheitszustand
Fallzahl *N*^a^	10.117	10.117	13.710	13.733	13.974
M (SD) Outcome	50,01 (9,96)	50,32 (9,87)	7,58 (1,65)	7,07 (2,16)	3,61 (0,98)
Wertebereich Outcome (in der Stichprobe)	3,89; 77,58	11,84; 74,22	0; 10	0; 10	1; 5
Intercept	21,82 (1,83)	28,00 (1,55)	2,59 (0,21)	3,49 (0,25)	1,59 (0,11)
*Kontrollvariablen:*					
SF-12 psychische Gesundheit (2018)	0,37 (0,01)***	/	/	/	/
SF-12 körperliche Gesundheit (2018)	/	0,43 (0,01)***	/	/	/
Lebenszufriedenheit (2019)	/	/	0,46 (0,01)***	/	/
Zufriedenheit mit Gesundheit (2019)	/	/	/	0,46 (0,01)***	/
Allg. Gesundheitszustand (2019)	/	/	/	/	0,48 (0,01)***
Tage seit erstem Lockdown	0,01 (0,001)***	−0,002 (0,001)^b^	0,0003 (0,0002)	−0,0003 (0,0002)	0,000 (0,000)
*Prädiktoren:*					
Alter	0,07 (0,01)***	−0,10 (0,01)***	0,004 (0,001)***	−0,011 (0,001)***	−0,01 (0,0006)***
Geschlecht (männlich)	1,08 (0,20)***	0,45 (0,16)**	−0,08 (0,03)**	0,06 (0,03)^b^	0,07 (0,01)***
Nettoäquivalenzeinkommen (logarithmiert)	0,84 (0,22)	0,90 (0,19)***	0,19 (0,03)***	0,13 (0,03)***	0,09 (0,01)***
Arbeitsstunden/Woche	−0,003 (0,011)	0,01 (0,01)	−0,002 (0,001)	0,002 (0,002)	0,0001 (0,001)
Anzahl Kinder bis 13 J. im Haushalt	0,05 (0,10)	0,15 (0,08)^b^	0,04 (0,01)**	0,02 (0,02)	0,01 (0,007)*
*Migrationshintergrund (Referenz* *=* *kein Migrationshintergrund)*					
Direkt	0,43 (0,26)^b^	−1,01 (0,21)**	0,16 (0,03)***	0,14 (0,04)***	0,03 (0,02)^b^
Indirekt	−0,08 (0,40)	−0,07 (0,31)	0,09 (0,05)^b^	0,05 (0,06)	−0,02 (0,03)^b^
Beruflich selbstständig	−0,47 (0,41)	0,36 (0,32)	−0,07 (0,04)^b^	0,05 (0,05)	0,01 (0,02)
Chronische Erkrankung	−1,77 (0,21)***	−3,81 (0,18)***	−0,31 (0,03)***	−0,77 (0,04)***	−0,36 (0,02)***
*Körpergewicht (Referenz* *=* *normalgewichtig und darunter)*					
Übergewicht	0,28 (0,21)	−0,98 (0,17)***	0,02 (0,03)	−0,09 (0,03)**	−0,07 (0,01)***
Adipositas	−0,11 (0,26)	−2,00 (0,21)***	−0,07 (0,04)^b^	−0,36 (0,05)***	−0,16 (0,02)***
Behinderung	−1,54 (0,40)***	−2,25 (0,30)***	−0,14 (0,06)*	−0,45 (0,07)***	−0,16 (0,03)***
Verheiratet	0,03 (0,26)	0,11 (0,20)	0,10 (0,03)**	0,02 (0,04)	0,02 (0,02)
Ostdeutschland	−0,08 (0,22)	0,06 (0,18)	−0,04 (0,03)	0,03 (0,04)	0,04 (0,02)**
Alleinerziehend	−2,03 (0,60)***	0,09 (0,50)	−0,22 (0,08)*	−0,10 (0,09)	0,01 (0,04)
Singlehaushalt	−0,58 (0,32)^b^	1,18 (0,24)***	−0,07 (0,04)	0,07 (0,05)	0,07 (0,02)***
*Bildung: CASMIN-Klassifikation (Referenz* *=* *niedrig)*					
Mittel	−0,09 (0,24)	0,89 (0,20)***	0,03 (0,03)	0,06 (0,04)	0,04 (0,02)*
Hoch	−0,32 (0,28)	1,46 (0,23)***	−0,01 (0,04)	−0,03 (0,05)	0,06 (0,02)**
*Erwerbsstatus (Referenz* *=* *Vollzeit)*					
Teilzeit	−0,10 (0,30)	0,14 (0,24)	−0,07 (0,04)	0,08 (0,05)	0,01 (0,02)
Nicht erwerbstätig	−0,88 (0,28)***	−0,78 (0,22)***	−0,09 (0,04)*	−0,01 (0,04)	−0,04 (0,02)*
Sonstiges	0,23 (0,40)	−0,26 (0,30)	−0,07 (0,05)	−0,01 (0,06)	−0,01 (0,03)
*Modellfit*					
F (df)	F_(28, 10088)_ = 73,47***	F_(28, 10088)_ = 330,91***	F_(28, 13681)_ = 116,03***	F_(28, 13704)_ = 289,37***	F_(28, 13945)_ = 413,87***
R^2^	0,194	0,476	0,279	0,406	0,456
R^2^ ohne Kontrolle der Ausgangswerte (Differenzwerte)	0,012	0,009	0,014	0,007	0,007
RMSE	8,959	7,153	1,405	1,666	0,722
*Vergleichsergebnisse der Random Forests*					
Vorhersagefehler (Test Error, RMSE)	10.912	8,425	1,644	1,938	0,843
R^2^ (Testdaten)	0,016	0,010	0,011	0,007	0,004
*Vorhergesagte Outcomes*					
M (SD)	−0,771 (1,274)	−0,290 (0,715)	−0,002(0,221)	−0,011(0,13)	−0,002(0,059)
Range	−5,075; 5,275	−2,982; 3,973	−1,067; 1,400	−0,556; 0,678	−0,328; 0,256

*CASMIN* Comparative Analysis of Social Mobility in Industrial Nations, *M* arithmetisches Mittel, *RMSE* Root Mean Squared Error, *SD* Standardabweichung, *s.* *e.* robuster Standardfehler, *SF-12* Short-Form-Health Survey

^a^Fehlende Werte in Prädiktorwerten wurden mit Hilfe des R‑Paketes *missRanger* imputiert, welches Random Forests zur Vorhersage der Werte nutzt; fehlende Werte im Outcome wurden nicht geschätzt, sondern die entsprechenden Fälle ausgeschlossen; ^b^ *p* ≤ 0,10

*** *p* ≤ 0,001; ** *p* ≤ 0,01; * *p* ≤ 0,05

**Table 4:** 

	∆ Psychische Gesundheit (SF-12)	∆ Körperliche Gesundheit (SF-12)	∆ Allg. Gesundheitszustand	∆ Lebenszufriedenheit	∆ Zufriedenheit mit Gesundheit
Einkommen pro Kopf	2	1	1	1	1
Zeit seit Lockdown	1	2	2	2	2
Arbeitsstunden/Woche	3	3	3	3	3
Alter	4	4	4	4	4
Anzahl Kinder bis 13 J.	5	5	5	5	5
Chronische Erkrankung	–	6	7	9	6
Übergewicht	–	–	6	8	8
Adipositas	7	7	8	9	–
Bildung – gering	9	–	–	6	–
Bildung – mittel	12	–	–	–	–
Bildung – hoch	–	–	–	–	7
Bildung – keine Angabe	6	8	–	7	–
Nicht erwerbstätig	8	–	–	–	–
Migration – direkt	–	–	–	–	–
Migration – indirekt	10	–	9	–	–
Ostdeutschland	–	9	–	–	–
Alleinerziehend	11	–	–	–	–

∆ vorhergesagt wurden jeweils die Differenzwerte der Outcomes zum subjektiven Wohlbefinden; die Rangfolgen beruhen auf der in den Random Forests durch *Ranger* ermittelten Werten der Variablen-*Importance*; angegeben sind je Outcome die Variablen mit Werten über dem 75 %-Perzentil

Als wichtigste Splitvariablen für alle Wohlbefindensmaße erwiesen sich Nettoäquivalenzeinkommen, die bisherige Dauer der Pandemie, Anzahl der Arbeitsstunden, das Alter sowie die Anzahl der Kinder im Haushalt. Weitere Variablen waren jeweils nur für einige Outcomes relevant. Zum Beispiel spielte das Vorliegen einer chronischen (Vor‑)Erkrankung eine Rolle hinsichtlich der berichteten Veränderung des körperlichen Wohlbefindens, des allgemeinen Gesundheitszustands und der Zufriedenheit mit der Gesundheit, jedoch weniger hinsichtlich des psychischen Wohlbefindens. Auch Übergewicht bzw. starkes Übergewicht (Adipositas) fand sich mehrfach unter den wichtigsten Variablen wie auch der Bildungsgrad. Vereinzelt waren auch Migrationshintergrund, Wohnort in den östlichen Bundesländern sowie der Status „alleinerziehend“ unter den wichtigsten Merkmalen.

#### Allgemeines lineares Modell

In Tab. 3 stellen wir die Effekte in Form von Koeffizienten linearer Regressionsmodelle dar. Die Modelle stellen den Wohlbefindenswert nach Lockdown adjustiert um den Ausgangswert dar, um die Veränderung abzubilden. Hinsichtlich der Varianzerklärung ist zu beachten, dass diese sich hier auf das Outcome nach Lockdown, inkl. der durch die Baseline-Werte erklärten Unterschiede, bezieht. Zur besseren Vergleichbarkeit mit den Ergebnissen der Random Forests geben wir R^2^ ergänzend für das Modell der Differenzwerte an. Außerdem finden sich in Tab. 3 unten die Maße für Varianzerklärung und Vorhersagefehler der Random Forests.

Mit allen Veränderungen im Wohlbefinden signifikant assoziiert war das Alter der Befragten. Es zeigten sich eher positive Veränderungen mit zunehmendem Alter für das psychische Wohlbefinden (SF-12 psychisch und Lebenszufriedenheit) und negative Veränderungen hinsichtlich der körperlichen Gesundheit (SF-12 körperlich, allgemeiner Gesundheitszustand und Zufriedenheit mit der eigenen Gesundheit). Die Koeffizienten deuten auf geringe Unterschiede hin (z. B. wäre ein Anstieg im Alter um 10 Jahre nach dem Modell mit einem Anstieg des SF-12-Werts für die mentale Gesundheit um ca. 0,7 Punkte assoziiert, was weniger als 1/10 SD entspricht).

Männliche Befragte gaben fast durchgehend günstigere Werte an als weibliche. Positiv mit den meisten Outcomes assoziiert war auch das Einkommen. Verglichen mit Personen ohne Migrationserfahrung gaben eingewanderte Personen überwiegend positivere Veränderungen in den Gesundheitsvariablen an.

Durchweg ungünstigere Outcomes berichteten Personen mit chronischen Erkrankungen, wobei der Rückgang in der körperlichen Gesundheit (SF-12) am deutlichsten ausfiel. Signifikant deutlicher war der Rückgang in allen Wohlbefindensvariablen auch bei Menschen mit Behinderungen. Von Übergewicht oder Adipositas betroffene Personen berichteten stärkere Rückgänge in der körperlichen Gesundheit, nicht aber dem psychischen Wohlbefinden.

Nicht regulär Erwerbstätige zeigten fast durchgängig schlechtere Outcomes als die Referenzgruppe der Vollzeitbeschäftigten. Weitere Merkmale waren weniger konsistent oder gar nicht signifikant mit Veränderungen im Wohlbefinden assoziiert.

Die erklärte Varianz im subjektiven Wohlbefinden nach Eintreten der Pandemiesituation variiert zwischen etwa 19 % für die psychische und ca. 47,5 % für die körperliche Gesundheit bei Einbezug der Ausgangswerte. Ein Vergleich mit einem Modell der Differenzwerte zeigt jedoch, dass das aktuelle subjektive Wohlbefinden vorwiegend durch die Ausgangswerte vor Lockdown erklärt wird. Ohne Adjustierung bewegt sich der erklärte Varianzanteil der Differenzen für alle Outcomes um die 1 %-Marke. Dass dieser Anteil in den Random-Forest-Modellen nicht höher ausfällt, zeigt, dass der Einbezug von Wechselwirkungen hier keinen Vorteil bringt.

Insgesamt erwiesen sich damit vor allem Merkmale zum subjektiven Wohlbefinden sowie das Vorliegen von Behinderungen oder chronischen Erkrankungen vor Lockdownbeginn als entscheidend für das subjektive Wohlbefinden im Verlauf des ersten Pandemiejahres.

## Diskussion

Hauptziel unserer Analyse war die Identifizierung von Risikogruppen für ein verringertes Wohlbefinden infolge der COVID-19-Pandemie und assoziierter Maßnahmen, wobei intersektionale Effekte explizit berücksichtigt werden sollten. Dazu wurden verschiedene Maße zum subjektiven Wohlbefinden in ihrer Veränderung beim Übergang in die Phase pandemiebedingter Einschränkungen betrachtet und es wurde durch ein exploratives Machine-Learning-Verfahren nach Gruppen mit unterschiedlichen Verläufen gesucht.

Trotz Verwendung von Random Forests mit einem Fokus auf die Optimierung der Vorhersage und Berücksichtigung statistischer Interaktionen zeigen die Ergebnisse insgesamt eine nur sehr geringe Erklärung von Veränderungen im Wohlbefinden durch die einbezogenen sozialen Kategorisierungsmerkmale. Es ergaben sich damit keine klar abgrenzbaren homogenen Risikogruppen. Insbesondere intersektional definierte Risikogruppen wurden nicht identifiziert.

Dieses Ergebnis bestätigte sich in einem linearen Regressionsmodell. Ohne Kontrolle für die Werte vor der Pandemie erklärte auch dieses Verfahren nur einen vergleichbar geringen Varianzanteil in den Differenzwerten, d. h. Veränderungen im Wohlbefinden. Eine Identifizierung von Risikogruppen lässt sich durch diese Ergebnisse nicht mit ausreichender Sicherheit vornehmen.

Auch die Höhe der Regressionskoeffizienten, die die Effekte potenzieller Risikofaktoren darstellen, fällt überwiegend gering aus. Die deutlichsten Unterschiede zeigten sich für Ausprägungen gesundheitlicher Merkmale vor Pandemiebeginn. Insbesondere chronische Vorerkrankungen gingen mit einer stärkeren Reduktion des subjektiven Wohlbefindens einher, wobei v. a. körperliche Gesundheitsaspekte betroffen waren. Ähnlich fallen die Ergebnisse bei Vorliegen einer Behinderung aus. In geringerem Ausmaß galt dies außerdem für (starkes) Übergewicht. Hier wäre jeweils sinnvoll, in weiteren Untersuchungen die Mechanismen näher zu beleuchten, welche zu diesem Rückgang beigetragen oder aber ihm entgegenwirkt haben.

Nur bei Einbezug der Ausgangswerte des subjektiven Wohlbefindens ließ sich ein nennenswerter Anteil an Varianz im Wohlbefinden nach Lockdownbeginn erklären. Das heißt, auch hier bestimmt der Ausgangszustand das Wohlbefinden nach Einsetzen der NPI. Dabei wurden diese Werte in die Regressionsmodelle einbezogen, um für Unterschiede in den Ausgangswerten zu adjustieren und dadurch Effekte der Gruppierungsvariablen auf *Veränderungen *abzubilden. Ein deutlicher Zusammenhang zwischen verschiedenen Werten einer Person über die Zeit ist dabei durchaus üblich.

Die beobachtete gesundheitliche Gefährdung durch Vorerkrankungen, Behinderungen, ein höheres Alter oder starkes Übergewicht deckt sich mit Beobachtungen höherer Risiken für Infektionen und schwere Verläufe [12]. Hier sind also die gleichen Merkmale mit direkten wie auch indirekten Pandemiefolgen assoziiert. Übereinstimmend mit Analysen des Infektionsgeschehens [8] berichteten Befragte mit höherem Bildungsabschluss eine geringfügig bessere körperliche Gesundheit.

Einen stärkeren Rückgang der psychischen Gesundheit fanden wir im Regressionsmodell bei Alleinerziehenden. Hier könnte sich eine höhere Belastung durch die Kinderbetreuung im psychischen Wohlbefinden niederschlagen. In der Literatur wird vor allem die Mehrbelastung von Müttern durch die Kinderbetreuung im Lockdown thematisiert [13].

Erstaunlich ist, dass die vielfach nachgewiesenen gesundheitlichen Benachteiligungen, die mit einem geringen sozioökonomischen Status einhergehen, sich in unseren Ergebnissen zum subjektiven Wohlbefinden kaum widerspiegeln. Es finden sich insbesondere auch solche Gruppen nicht klar wieder, die laut anderen Veröffentlichungen besonders stark von wirtschaftlichen Folgen des Lockdowns betroffen waren, z. B. selbstständige Frauen [17, 30], Erwerbstätige mit geringem Bildungsniveau und Einkommen [14] sowie Menschen mit Migrationserfahrung [15].

Eine mögliche Erklärung wäre, dass sich pandemiebedingte Belastungen erst mit zeitlicher Verzögerung im Wohlbefinden zeigen und diese Effekte hier noch nicht erfasst wurden, sich aber in nachfolgenden Erhebungen zeigen. Sollte dies nicht zutreffen, wäre es aufschlussreich zu untersuchen, welche Ressourcen einer Beeinträchtigung im Wohlbefinden in Bevölkerungsgruppen mit größeren wirtschaftlichen Einbußen entgegenwirken.

Mena und Bolte [47] fanden in einer Entscheidungsbaum-Analyse der Studie „Gesundheit in Deutschland aktuell“ (GEDA) aus 2009, dass Gruppen mit schlechter mentaler Gesundheit sich v. a. durch geringe soziale Unterstützung und hohe Belastungen durch Haushalt oder Pflege‑/Betreuungsaufgaben identifizieren ließen. Dies betraf häufiger Frauen. Sie verweisen auf erklärende Mechanismen für gesundheitliche Ungleichheiten, während soziokulturelle und ökonomische Variablen praktisch nicht als Variablen für Gruppenunterscheidungen bedeutsam waren. Eine Befragung zur retrospektiven Beurteilung der Lebenszufriedenheit vor, während und nach dem ersten Lockdown zeigte ebenfalls, dass die Beurteilung der eigenen Lebenssituation während der Pandemie (z. B. bezüglich sozialer Kontakte sowie Familien- und Arbeitsleben) Veränderungen der Lebenszufriedenheit über soziodemografische Merkmale hinaus erklären konnte [48]. Da in unserer Auswertung durchaus auf individueller Ebene Verläufe mit deutlichen Veränderungen im Wohlbefinden beobachtet wurden, könnten Unterschiede in Belastungen und Ressourcen, welche nicht direkt mit sozialen Kategorisierungen zusammenhängen, entscheidend für die Erklärung von Wohlbefindensveränderungen in der Pandemie sein.

Sehr ähnliche Unterschiedsmuster wie wir berichten Eicher et al. [18] aus einer App-Befragung während der Pandemie in Abhängigkeit von sozialen Merkmalen wie Alter, Geschlecht, Bildung, Erwerbssituation sowie chronischen Erkrankungen. Die Varianzaufklärung für körperliche und psychische Wohlbefindensaspekte erreichte ca. 20–30 % in der Erhebung im zweiten Halbjahr 2020. Entscheidend ist, dass dabei interindividuelle Unterschiede erklärt wurden, während wir uns auf Veränderungen beziehen, bei denen Unterschiede im Ausgangsniveau egalisiert wurden.

Bedeutsame Wechselwirkungen im Sinne intersektionaler Effekte konnten wir nicht identifizieren. Dies widerlegt intersektionale Ansätze nicht. Zum einen benennen diese keine spezifischen Intersektionen, welche hinsichtlich bestimmter Gesundheitsmaße betroffen sind. Zum anderen stellen sie keine falsifizierbare Theorie dar, sondern eine kritische Perspektive auf soziale Ungleichheit und Benachteiligung [49]. Da auch die Haupteffekte der Sozialvariablen nur geringe Gruppenunterschiede aufzeigten, stellen die Ergebnisse unseres Erachtens auch nicht die grundsätzliche Untersuchbarkeit von Intersektionalität in quantitativen Studien infrage.

Jedoch scheinen Maßnahmen zur Stabilisierung der Gesundheit für spezifische Risikogruppen anhand dieser Ergebnisse kaum gerechtfertigt. In einer Auswertung eines großen schwedischen Gesundheitssurveys (vor der COVID-19-Pandemie) definierten Wemrell et al. [50] Intersektionalität über eine Kombination von Migration, Geschlecht und Einkommen als Strata und fanden trotz sozialem Gradienten keine gute Vorhersage von Gruppen mit schlechtem subjektiven Gesundheitszustand und keine Verbesserung durch Einbezug der intersektionalen Strata gegenüber einzelnen Prädiktoren. Sie warnen, dass Public-Health-Interventionen für Risikogruppen die Gefahr beinhalten, entgegen ihrer Intention bestimmte Personen zu stigmatisieren und zu benachteiligen, wenn die identifizierten Risikogruppen nicht homogen sind.

### Stärken und Limitationen

Stärken unserer Analyse bestehen in der großen Stichprobe und einer Vielzahl potenziell relevanter Indikatoren durch Nutzung des SOEP. Insbesondere erlaubt der Datensatz eine Analyse individueller Veränderungen im Längsschnitt und es besteht die Möglichkeit, Entwicklungen zukünftig weiterzuverfolgen. Außerdem berücksichtigen Random Forests nicht-lineare und nicht-additive Beziehungen in der Exploration potenzieller Risikogruppen und sind geeignet, im Sinne intersektionaler Ansätze verschiedenste Konstellationen von Merkmalen bei der Vorhersage einzubeziehen, ohne diese in ihrem Wechselwirken vorher genauer zu definieren.

Unser Vorgehen hat aber auch Limitationen. Das subjektive Wohlbefinden hängt von diversen Einflüssen sowie persönlichen Standards ab. Es ist nicht auszuschließen, dass systematische Unterschiede im Antwortverhalten zwischen den untersuchten Bevölkerungsgruppen bestehen. Betrachtet wurden hier außerdem allgemeine soziale Kategorisierungen, ohne nach pandemiebedingten Aspekten zu fragen. Dies ist auch ein möglicher Grund für recht geringe Variationen im Outcome. Allerdings schien die Beschränkung auf allgemeine, einfach verfügbare Indikatoren der Fragestellung angemessen, da spezifischere Maße vor oder während Krisensituationen wie einer Pandemie nicht breit verfügbar vorliegen. Sie sind damit weniger geeignet für die Identifikation von Risikogruppen auf Bevölkerungsebene.

Die Unterscheidung zweier Zeitperioden vor und nach dem Beginn des ersten Lockdowns ist möglicherweise nicht differenziert genug, um zeitliche Veränderungen der Outcomes angemessen zu erfassen. Zum Beispiel veränderten sich sozioökonomische Effekte für direkte Gesundheitsfolgen deutlich über die Zeit, gerade zu Beginn der Pandemie [3, 7]. Auch aus diesem Grund haben wir den zeitlichen Verlauf in der Auswertung berücksichtigt. Dabei ergaben sich bislang keine Hinweise auf zeitlich verzögerte Rückgänge im Wohlbefinden.

## Fazit

Der Gesundheitszustand vor Pandemiebeginn scheint für das subjektive Wohlbefinden bedeutsamer zu sein als demografische und sozioökonomische Kategorisierungsmerkmale. Letztere ermöglichen es bislang nicht, Bevölkerungsgruppen mit erhöhtem Risiko für ein verringertes Wohlbefinden bei Einsetzen der pandemiebedingten Einschränkungen zu identifizieren. Insbesondere ließen sich auch keine Konstellationen von Risikovariablen identifizieren, deren intersektionales Zusammenwirken Ungleichheiten im Wohlbefinden erklärt. Aus der Tatsache, dass der Ausgangszustand eine Rolle spielt, lässt sich aber folgern, dass die Förderung eines hohen Wohlbefindens insgesamt zur Resilienz der Bevölkerung in Krisensituationen beitragen könnte.

## Supplementary Information





## Footnotes

1 https://www.diw.de/en/diw_01.c,789785.en/documentation_of_soep-core__soep_survey_papers.html (SOEP Survey Papers; 1183: Series D—Variable Description and Coding/2022); siehe auch https://paneldata.org.
