# Supplementary material for: Consequences of the COVID-19 pandemic: Are there risk groups for reduced subjective well-being after the first lockdown?
Source: Bundesgesundheitsblatt Gesundheitsforschung Gesundheitsschutz. 2023 Jul 20;66(8):824–34. [Article in German] doi: 10.1007/s00103-023-03737-w (PMC10371891; doi:10.1007/s00103-023-03737-w)
Supplement: Supplementary file 1 [file 103_2023_3737_MOESM1_ESM.pdf]

Beschreibung der Methode „Random Forests“

„Random Forests“ gehören zu den „Ensemble learners“. Sie bestehen aus einer Zusammenfassung vieler Entscheidungsbäume zu einem „Wald“.

Das Grundprinzip von Entscheidungsbäumen besteht darin, dass ein in einem vorliegenden Datensatz bekanntes Outcome möglichst korrekt aus vorhandenen Variablenausprägungen vorhergesagt wird. Das Outcome kann kategorial (Klassifikation) oder metrisch (Regression) skaliert vorliegen. Aus einer Menge an potenziellen Vorhersagevariablen wird in jedem Schritt diejenige Variable ausgewählt, welche den Vorhersagefehler minimiert. Dazu wird ein Variablen*split* verwendet: Es wird der Schwellenwert ermittelt, welcher am besten den Fehler minimiert.

Dadurch entstehen jeweils zwei Gruppen: über und unter dem Schwellenwert. In beiden Gruppen wird im nächsten Schritt jeweils erneut derjenige Variablensplit ermittelt, der nun die beste Vorhersage innerhalb jeder Gruppe ermöglicht. Dies wird fortgesetzt, bis ein Abbruchkriterium erreicht wird (eine Mindestanzahl an Fällen je Gruppe oder eine nur noch sehr geringe Verbesserung durch weitere Unterteilungen).

Es entstehen also bei jedem Split Verzweigungen. Jede neue Beobachtung kann anhand der gemessenen Merkmalsausprägungen entlang dieser Baumstruktur klassifiziert werden. An jeder Verzweigung entscheidet sich aufgrund einer Variablenausprägung, welchem Pfad weiter zu folgen ist. Allerdings optimieren Entscheidungsbäume immer nur den aktuellen Split (‚greedy algorithm‘), was sich auf den weiteren Aufbau des Baumes negativ auswirken kann, da sich Fehler bei frühen Splits fortsetzen und kleine Veränderungen in den Daten so zu sehr unterschiedlichen Bäumen führen können.

Bei Random Forests (zuerst [42]) werden daher anhand von (zufälligen) Bootstrap-Stichproben der Ausgangsdaten viele solcher Entscheidungsbäume erstellt und je Split nur eine Teilmenge aller Splitvariablen berücksichtigt. Die resultierenden Vorhersagen werden dann gemittelt. Als Vorhersagewert verwendet wird die häufigste Kategorie bzw. der Mittelwert in der entsprechenden Gruppe. Dieses Vorgehen führt i.d.R. zu sehr viel besseren Vorhersagen als einzelne Entscheidungsbäume, allerdings auch zu einer weniger guten Interpretierbarkeit der Ergebnisse, da die anschauliche Baumstruktur verloren geht (s. z.B. [41]).

Für jeden Split wird dabei nur eine zufällig ausgewählte Teilmenge aller potenziellen Splitvariablen verwendet. Durch diese Zufallsauswahl wird verhindert, dass die zusammenzufassenden Bäume des „Waldes“ aufgrund der jeweils gleichen Prädiktoren korreliert sind. Dadurch wird die Zuverlässigkeit der durchschnittlichen Vorhersagen nochmals erhöht[1].

Bei der Entwicklung eines Vorhersagemodells werden vorhandene Daten i.d.R. in Trainings- und Testdaten aufgeteilt. Der Trainingsdatensatz wird genutzt, um das beste Vorhersagemodell zu entwickeln, was auch die optimale Auswahl der „Tuningparameter“, bei „Random Forests“ v.a. die Anzahl pro Split verwendeter Variablen, beinhaltet. Da das Interesse darin liegt, ein generalisierbares Vorhersagemodell zu entwickeln, welches auch außerhalb der Stichprobe funktioniert, d.h. „Overfitting“ zu vermeiden, wird final der Testfehler an den noch nicht verwendeten Testdaten ermittelt.

---

[1] Ansonsten würden bestimmte Prädiktoren zu sehr dominieren, die in den meisten Trees ausgewählt werden und damit zu korrelierten Trees führen. Dann führt die Durchschnittsbildung nicht mehr zu einer effektiven Reduktion der Variabilität [42, S. 345].

Es besteht die Möglichkeit, die durch das Random-Forest-Verfahren unterschiedenen Gruppen hinsichtlich der vorhergesagten Outcomewerte sowie der Zugehörigkeit zu Prädiktorkategorien zu beschreiben. Weiterhin lässt sich ein repräsentativer Entscheidungsbaum aus dem Wald extrahieren.

Bei einem Vergleich von „Random Forests“ und einem allgemeinen linearen Modell hinsichtlich der Bedeutung einzelner Merkmale für die Vorhersage ist mit zu berücksichtigen, dass die Variablen in beiden Verfahren anhand unterschiedlicher Kriterien betrachtet werden. Auch sind die Regressionskoeffizienten im allgemeinen Linearen Modell jeweils für alle andere Prädiktoren adjustiert.

Unter den wichtigsten Variablen vorne stehen oft, wie auch in unserem Modell, kontinuierliche Prädiktoren (Einkommen, Zeit, Anzahl, Alter). Dies verwundert insofern nicht, als diese differenzierten Werte mehr Information enthalten, jeweils mehrfache Splits ermöglichen und damit häufiger aufgeteilt werden können, während die dummykodierten kategorialen Variablen jeweils nur eine Unterteilung erlauben. Der Variablenimportancewert ergibt sich aus der über alle Splits erreichten Reduktion der Residualquadratsumme. Die Anzahl der Verzweigungen, in denen eine Variable verwendet wird, kann also eine Rolle spielen. Ähnlich ist allerdings auch in den verwendeten Regressionsmodellen die erklärte Varianz für stärker differenzierte Prädiktoren potenziell größer.
